# Comparative Analyses of the Dominant and Non-Dominant Upper Limbs during the Abduction and Adduction Motions

**Published:** 2019-10

**Authors:** Haemi JEE, Jaehyun PARK

**Affiliations:** 1. Department of Physical Therapy, Namseoul University, Cheonan-si, Korea; 2. Department of Information and Communication, Inha University, Incheon, Korea

**Keywords:** Asymmetry, Dominance, Upper limb, Kinematics, Bilateral motion

## Abstract

**Background:**

Asymmetry in repeated motion may lead to dyskinesia through imbalance in the involved musculoskeletal structures. The dominance sides are also involved greater movement involvement over the non-dominant sides. The upper limbs with multiple joints and largest range of motion are prone for unsynchronized coordination. Natural movement analysis is required for application to everyday activities.

**Methods:**

Thirty participants were first recruited from Inha University, Incheon, Korea in 2019. Twenty subjects were assessed for comparisons of asymmetrical motion between the dominant and non-dominant arms during the abduction and adduction lateral raises after excluding ten subjects for shoulder pain and left-handedness.

**Results:**

The abduction and adduction motions of the bilateral arms were compared for the angular locations, velocity, and acceleration for every 10 degrees. The angular locations of the dominant side occurred significant earlier in the initial (10°, 20°, 30°) phase and later in the last (10°, 20°) phase of abduction and adduction in comparison to the non-dominant side (*P*<.05). The angular accelerations of the dominant side were also significantly greater during the initial phase (0°, 10°, 30°) and last phase (0°, 10°, 30°) (*P* <.05). The angular velocities were significantly greater during the later phase (40, 50, 60°) of abduction (*P* <.04).

**Conclusion:**

Comparative dominant side indicated more controlled movements through the range of motion with greater stability in angular acceleration and deceleration especially during the initial and last phase of abduction and adduction, respectively. Training for control of the specific angular points should be considered during abduction and adduction motions to prevent asymmetry of the bilateral arms.

## Introduction

Early evaluation of abnormal posture or movement can detect inefficiency in muscular function for preventing progress into musculoskeletal pathology (1–3). Various assessment methods can be utilized to assess abnormality. Various methods including photography, force platform, Computed tomography (CT), magnetic resonance imaging (MRI), X-ray, and 3-D motion analysis system, inertial sensor, and optical marker tracking have been utilized to assess the postural or kinematic imbalance (1, 4–6). However, movement assessment results have been questioned for their assessment inaccuracy (1, 4, 5). These commonly assessment methods have limitations of assessing photographed pictures or acquiring radiation exposure (1, 5). In addition, previous methods are limited to a certain fixed space for assessing natural movements or kinematics (4). That is, assessments must be conducted in certain space with devices fixed in specific location. In order to observe possible movement imbalance that may further progress to musculoskeletal pathology, naturally performed movements conducted in everyday situation should be assessed (7, 8).

One of the common assessment practices has been to compare dynamic kinematics of the symmetric sides such as compare of the left and right upper or lower limbs. Identical musculoskeletal structures exist for both the ipsilateral and contralateral sides. Difference in strength, musculature, or usage between the symmetric sides may promote musculoskeletal dysfunction and further progress into pathology. Dominant and nondominant side comparisons have been previously conducted to compare and observe the abnormality between the ipsilateral and contralateral sides for possible imbalance (9). Due to the repeating nature of the forceful movements specific to the type of sport of the athletes, difference in the kinematics of the dominant and nondominant sides have been reported in athletes or those involved in physical activity (7, 10). Excessive usage of one particular side and segment has been reported to lead to dysfunctional asymmetry (5, 7, 10).

Among the symmetrical bodily sides, the upper extremities with the shoulder joints have been commonly observed for dyskinesia. The shoulder with 3 bones and 4 joints, the antagonist, agonist, and synergist muscles with scapula create the greatest range of motion and complexity in movements among all bodily joints (4). In order to assess the delineate abnormality of the shoulder, although absolute evaluation in commonly performed, the functionality of one side is commonly compared with the contralateral side (5).

Despite the importance of accurate assessment in the upper extremities with the shoulder complex, synchronized assessment was rarely conducted during kinematic conditions. Therefore, in order to compare the natural functional motions of the upper extremities with the shoulder complex, this study utilized dime-sized 3-D motion sensors on each limb during repeated abduction and adduction motions. Positions by angle (°), angular velocity (deg/s), and angular acceleration (deg/s^2^) for each 10-degree position were compared between the dominant and non-dominant sides. Moreover, variability in angular velocity and acceleration were compared between the dominant and non-dominant sides.

## Methods

### Subjects

Thirty participants were first recruited from Inha University, Incheon, Korea in 2019. Prior to the tests, all participants were fully informed of the purpose and procedure of the experiment. Among the initial thirty participants, ten were excluded for left-handedness and shoulder pain. Twenty right-handed subjects aged between 20 to 28 years with mean age of 22.1 (±2.34) years and mean body mass index (BMI) of 22.7 (±2.34) kg/m^2^ participated in performing lateral raise with two dumbbells held on both hands. The subjects regularly participated in physical activity 2.3 (±2.23) days per week and 34.3 (±36.68) minutes per day on the average at a low intensity of 2.7 (±2.80) assessed by RPE. The pain scale of 4.0 (±8.53) %, disability scale of 1.4 (±2.4) %, and overall scale showed 2.4 (±4.04) %. The average dominant handedness score was 9.0 (±1.16) out of scales from −10 to 10. Negative values indicated left-handedness and positive values indicated right-handedness.

The study was approved by the Inha University Ethics Committee and performed according to the Declaration of Helsinki. The participants gave verbal and written informed content to participate in the study.

### Experimental procedure

Lateral raises were conducted in accordance with previous study (11). Borg scale of 0 to 10 was used to assess rate of perceived exertion (RPE) immediately after performing lateral raise for the exercise intensity (11, 12). Participants were asked to consume a small-sized meal with plenty of liquid 2 hours prior to the assessment. In addition, the participants were asked to restrain from physical activity one day prior to the testing.

### Questionnaires

General information such as weight (kg), height (g), and age (years) of the participants were acquire through pre-arranged questionnaires. In addition, general health status, exercise habit (exercise days per week, exercise time per day, and exercise intensity (Borg scale (0–10)), shoulder pain and disability status, and dominant hand assessment were done through questionnaires. Shoulder pain and disability was assessed with the Shoulder Pain Disability Index (SPADI) that expressed the results of total pain, total disability, and total score in percentage (%) (11). Since the minimum detectable change with 90% confidence interval due to shoulder pain and disability in 13 points for the SPADI, the participants with any type of significant shoulder pain was excluded from the study (13). Laterality quotient (L.Q) or dominant hand was assessed with the Edinburgh Inventory (14). Participants with scores more than 6 or above were included in the study to exclude mixed or left handers (15).

### Lateral raises

In order to exclude fatigue of non-dominant arm during the lateral raises, dumbbell weights of low intensity or less than 40% of 1-RM (repetition maximum) (16). 1-RM was derived from previous reported 1-RM calculation and trial-and-error methods (17). The subjects were asked to test the dumbbells ranging from 1 kg to 5 kg in weight prior to the actual assessment for prior familiarity. The subjects were shown and explained of the abduction-adduction movement procedure prior to holding the dumbbells. All exercises were performed in a slowly controlled manner. The subjects were told to stand erect with feet shoulder-wide apart and hold the dumbbells by both hands slightly abducted from the body. The subjects were then asked to lift (1.5 seconds) the dumbbells and abduct the shoulder joints until the upper arms were slightly above horizontal (minimum to maximum range of motion: 10° – 100°) and lower (1.5 seconds) without sudden jerks or acceleration. The abductions and adductions were performed in slow and controlled manner for twenty consequent repetitions or to voluntary failure. If the subjects were not able to compete another repetition through a full range of motion despite (less than 90 degrees abduction) a verbal encouragement, the incomplete repetition was not counted (12). Borg scale with range of 1 to 10 was used to assess the rate of perceived exertion (RPE) after performing the lateral raises.

### Motion assessment

Prior to performing the lateral raises, two devices were attached to both wrists near the distal radiuolnar joint on the backside of the wrist. The lateral raises were performed bilaterally and the kinetic information including synchronized position information by angle (°), acceleration (deg/s^2^), and velocity (deg/s) were collected and recorded every one-tenth of a second to a nearby computer real-time via Bluetooth. Results for the minimum angle (°) and maximum angle (°), and every 10 degrees (°) in between were collected. The minimum angle indicates the starting position when the upper limbs are near the center axis of the body and the maximum angle indicates the ending position when the upper limbs are horizontal to the shoulder line during the lateral raise movement. The lateral raises were continuously conducted at least twice, and the second lateral raises were selected for the analysis.

Experimental data were obtained by using CC2650 SensorTag from Texas Instruments equipped with accelerometer and gyroscope as sensor nodes (18). The feasibility assessment showed substantial to excellent correlations between 0.65 and 0.88 (*P*<0.05) with small effect sizes. To synchronize system time of both sensors, hardware interrupt method on each sensor was used. The sensor node transmits the measured data to the data processing node via BLE communication. The transmitted raw data were processed to estimate the motion using the Extended Kalman Filter (EKF) (19, 20).

### Statistical Analysis

The study sample size was determined using data from previous studies (n ≥ 10) that used the lateral raise. Prior to analytical assessments of data sets, the normality analysis was conducted using the Shapiro-Wilk test. Data were normally distributed. A pre-test of variance homogeneity was also conducted with the Levene's test. One-way ANOVA (analysis of variance) was conducted to determine the differences in variables between dominant and non-dominant arms. Results were presented as means ± standard deviations (SD). Statistical significance was accepted for the *P-*value of 0.05.

## Results

Two sets of assessed results from the abduction and adduction motion with dumb-bells and sensors on the proximal ends of the upper limbs were assessed. Data of the left upper limb was extracted with the matching data of the right upper limb from the initiation of abduction to the termination of adduction motion and for every 10 degree in between motions.

**Fig. 1:**
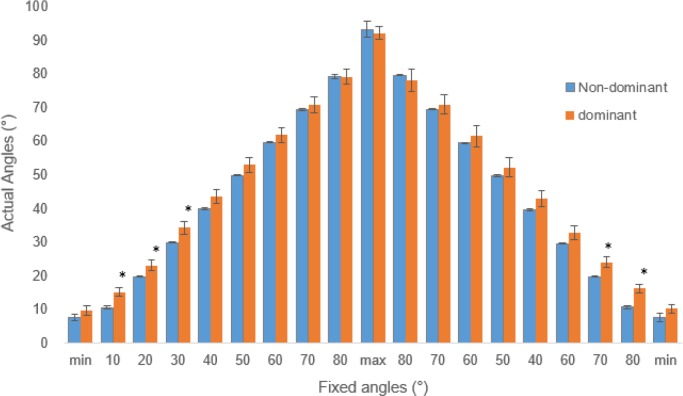
Comparisons of the positional changes during abduction and adduction motions of the dominant and non-dominant upper limbs

First, the actual angles of the left upper limb in comparison to the angles of the right upper limbs were compared and drawn as in Fig. 1. The initiating abduction positions for the dominant and non-dominant arms were 9.67±6.52° and 7.5±4.38° (*P*=.66), the maximally raised angles were 92.2±8.73° and 93.3±10.93° (*P*=.30), and the finishing or minimum angles were 10.3±5.53° and 7.7±5.55° (*P*=.16), respectively.

Significantly different angles during the initial phase of the abduction motion and the last phase of the adduction motion were shown. That is, during 10, 20, and 30 degree points, the dominant and non-dominant sides' movement angles were significantly different by (15.2±6.10° vs. 10.6±1.79°, *P*=.003), (23.1±7.00° vs. 19.8±.31°, *P*=.045), and (34.3±8.71° vs. 29.9±.81°, *P*=.003), respectively. Significant differences were also shown during the last phase (20°, 10°) of adduction (24.1±7.24° vs. 19.8±.33°, *P*=.016), and (16.3±5.52° vs. 10.7±.2.21°, *P*<.001), respectively.

In addition, the mean range of motions (minimum – maximum) during the abductions were 85.8±9.55° (67.9–101.8°) and 82.57±9.32° (63.6 – 98.4°) (*P*=.34) for the dominant and non-dominant arms, respectively. The mean range of motions during the adductions were 85.65±9.61° (67.9 – 102.0°) and 82.00±8.84° (68.58 – 98.05°) (*P*=.29) for the dominant and non-dominant arms, respectively, without significance.

**Fig. 2:**
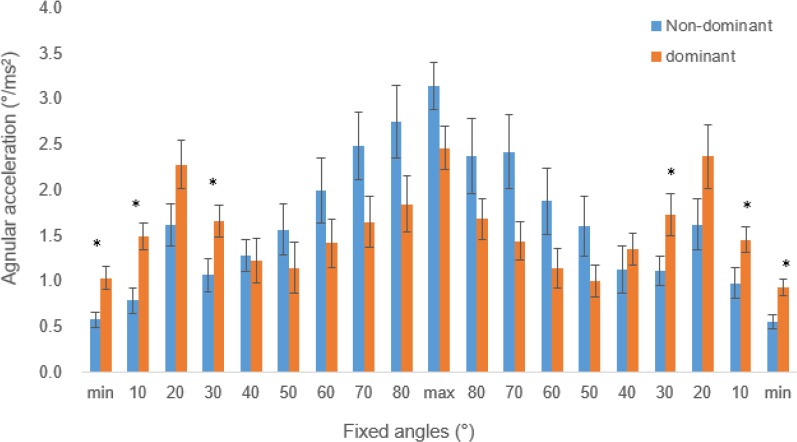
Comparisons of the acceleration changes during abduction and adduction motions of the dominant and non-dominant upper limbs

Second, the angular acceleration values were assessed for each of the selected angle points as shown Fig. 2.

The maximal angular acceleration and decelerations occurred during the initial (20°) and final (maximum angle) abduction points for both the dominant and non-dominant arms. The mean maximum accelerations at the maximum angle and 20° points during the abductions were significantly different with .97±1.20 and 2.36±1.00 deg/sec^2^ (*P*=.001) for the dominant and non-dominant arms, respectively. Maximal accelerations were also shown at the maximum angle and 20° during the adductions. The mean maximum accelerations at the maximum angle and 20° points during the adductions were significantly different with .91±1.27 and 2.21±1.13 deg/sec^2^ (*P*=.002) for the dominant and non-dominant arms, respectively. Moreover, significantly different accelerations were shown in the early phases of abduction and the last phases of adduction. Significance differences were .58±.37 vs. 1.04±.58 deg/s^2^, (*P*=.01), .79±1.04 vs. 1.49±.67 cm/ms^2^, (*P*=.001), and 1.07±0.81 vs. 1.66±.78 deg/s^2^, (*P*=.03) were observed at the initial phases, 10 and 30 degrees between the dominant and non-dominant sides, respectively.

Third, the angular velocity comparisons were also conducted for each of the selected angle points as shown in Table 1. Significant differences were shown during 40°, 50°, and 60° points during abductions.

**Table 1:** Comparisons of the velocity changes during abduction and adduction motions of the dominant and non-dominant upper limbs

***Fixed angles (°)***	***Dominant side velocity (°/ms)***	***Non-dominant side velocity (°/ms)***	***F***	**P *value***
min	1.19 (±.59)	1.41 (±.62)	.01	.26
10	1.23 (±.52)	1.26 (±.43)	3.39	.84
20	1.40 (±.60)	1.54 (±.52)	1.79	.42
30	1.26 (±.46)	1.53 (±.43)	.33	.06
40	1.08 (±.45)	1.44 (±.38)	.98	.01^*^
50	0.94 (±.41)	1.30 (±.40)	.06	.01^*^
60	0.89 (±.37)	1.16 (±.44)	.60	.04^*^
70	0.93 (±.45)	1.04 (±.47)	.00	.47
80	0.98 (±.59)	0.97 (±.53)	.07	.96
max	1.01 (±.45)	1.00 (±.54)	.10	.92
80	1.35 (±.48)	1.25 (±.60)	.14	.56
70	1.52 (±.55)	1.27 (±.56)	.84	.16
60	1.54 (±.61)	1.30 (±.56)	1.05	.22
50	1.55 (±.64)	1.30 (±.57)	2.20	.20
40	1.49 (±.67)	1.29 (±.54)	.72	.32
30	1.55 (±.57)	1.33 (±.54)	1.79	.22
20	1.59 (±.63)	1.44 (±.55)	3.53	.43
10	1.53 (±.75)	1.41 (±.52)	.69	.54
min	1.20 (±.49)	1.38 (±.46)	.39	.26

*: *P* <.05

Although the significances were not observed between the limbs, interesting findings were observed. The velocities at the 20-degree point of the initial abduction phase and last adduction phase showed the greatest velocities for both dominant (1.54±.52 deg/s) and non-dominant arms (1.40±.60 deg/s), respectively. In addition, the mean velocities of the dominant side were comparatively greater during the abduction phases and lesser during the adduction phases. Such asymmetric trend between abduction and adduction was similar for the angular locations. The dominant sides were raised before the non-dominant sides during abduction and the non-dominant sides were lowered before the dominant sides during adduction.

Last, the relative times of the dominant and non-dominant sides were recorded. The initiation of abduction (−15.7 vs. −21.2 ms), maximum point (129.6 vs. 130.7 ms), and termination of adduction (280.3 vs. 277.2 ms) were compared. In addition, the time durations (time duration at point/mean duration × 100) at the initiation of abduction (11.8 vs. 15.9 %), maximum point (15.3 vs. 16.2 %), and termination of adduction (17.9 vs. 15.6 %) were compared.

## Discussion

Asymmetry between the equivalent bodily sides may promote further dysfunctional kinematics and pathology. Therefore, it is important to detect asymmetry in functional movements during the early stage. Most of the people have a preferred side or dominant side. The dominant side is usually utilized over the non-dominant side during most of the functional movements including physical activity. This study compared dynamic kinematics of the upper extremities during abduction and adduction lateral raise movements with free weights on each arm of healthy young right-handed adults. The abduction and adduction motions were compared for the positions by angle of 10 degrees and the velocity and acceleration for each position (10°) between the dominant right arm and non-dominant left arm.

This study observed angle, velocity, and acceleration of synchronized lateral raises of dominant and non-dominant arms for kinematics differences. 10-degree angle comparison showed that the angular positions of the dominant and non-dominant arms were significantly different during the early abduction and late adduction phases despite similar mean values. The non-dominant side showed overall greater range of motion despite instructed maximum and minimum range of motion. That is, the non-dominant arms started the abduction movement and finished the adduction movement comparatively lower than the dominant arms (Fig. 1).

Mixed reports have been reported in terms of range of motion. In some studies, the maximum range of motions for various movements including abduction-adduction were reported to be similar regardless of sex or age (21, 22). However, the dominant side showed greater accuracy than the non-dominant side in this study. As for the minimum and maximum point comparisons, limited maximum (100°) and minimum (10°) range of motion were given prior to the assessment in this study. Although significances were not observed, the dominant arms initiated (9.67±6.52°) and terminated (10.3±5.53°) the abduction and adduction motions closer to the initial and final points. In addition, the overall greater range of motions (85.8±9.55° and 85.65±9.61°) closer to the range of motion indicated prior to the test.

Previous studies reported of greater rate usages or entropy of motion during random movements (15~20% during standing), the usage rates were similar during patterned motion such as walking (7, 23). Such results may be explained by the more torque-efficient movements made by the dominant arm than the contralateral side with greater accuracy and dynamic control (24). A previous study showed greatest muscle activities during the initial and final phases of the abduction and adduction movements, respectively (9).

Further accuracy motor control by the dominant side could be observed through angular acceleration comparisons. The dominant arms' mean angular acceleration was significantly greater during the initial and final phases of the abduction and adduction movements in this study, respectively (Fig. 2). On the other hand, the angular accelerations of the final and initial phases of the abduction and adduction movements were overall greater without significant differences for the non-dominant arms. Moreover, although the maximal angular accelerations were similar between the initial and final phases for both the dominant arms, the maximal angular accelerations for the non-dominant arms varied significantly. Such results indicate comparatively controlled motion created by the dominant arms in comparison to the non-dominant arms. Such controlled patterns by the dominant arms were similar in velocity changes throughout the range of motion. Refinement in motor control of the dominant side could be further observed by the degree of acceleration ranges. The differences in the maximal accelerations at 20 degrees and maximal angle of the non-dominant sides (2.36±1.00 and 2.21±1.13 deg/sec^2^) were more than twice that of the dominant sides. Such result was similar during the adduction phase. The dominant side show similar maximal acceleration results, indicating fine motor control ability.

Speed and accuracy of the dominant sides were both superior than the counterpart with speed-accuracy trade-off (25). The results could be interpreted that the dominant sides were able to partially sacrifice and control maximal potential speed to enhance accuracy in motion (25). Therefore, the dominant arms were able to more accurately control the degree of acceleration and deceleration for optimal control of the limb movement in comparison to the contralateral side. Nondominant sides have been reported to have less strength, power, refined motor control, and maximal speed (25, 26). Although lateral raises of the arms are simple to perform, the movement involves complex coordination of various areas of the body including muscles, ligaments, and tendons around the trunk, scapula, shoulders, elbows, and wrists (27, 28). The core segment of the body and the lower limbs are also involved for stability and isometric control during the upper limb motions which involve working with or against gravity (27, 28).

It is widely known that majority of athletes and participants of physical training experience asymmetry in motion and musculoskeletal involvement due to repeatedly utilized specific segments of the body (2, 3, 9, 25). However, due to repeated dominant usage of preferred sides without proper compensatory intervention, people acquire muscle imbalances or asymmetries at young ages (3, 29). Therefore, early detection for promoting muscular imbalance and asymmetry in motion should be carefully observed and intervened for proper compensatory action.

## Conclusion

Specific difference in movement patterns were observed between the dominant and non-dominant arms during abduction and adduction lateral raise motions. Refined motor control by the dominant sides was more prominent during the initial, maximal, finishing range of motion phases. The dominant sides were able to control range of motion, angular acceleration and velocity during critical phases. Specific points should be considered during lateral raises to gain and maintain muscular and movement balance between the dominant and non-dominant arms to avoid asymmetry in motion with corresponding musculatures.

## Ethical considerations

Ethical issues (Including plagiarism, informed consent, misconduct, data fabrication and/or falsification, double publication and/or submission, redundancy, etc.) have been completely observed by the authors.
